# Chitosan-based scaffold modified with D-(+) raffinose for cartilage repair: an in vivo study

**DOI:** 10.1186/s12952-014-0021-5

**Published:** 2015-01-14

**Authors:** Francesca Ravanetti, Carlo Galli, Edoardo Manfredi, Anna Maria Cantoni, Edoardo Scarpa, Guido Maria Macaluso, Antonio Cacchioli

**Affiliations:** Department of Veterinary Science, University of Parma, Via del Taglio 10, 43126 Parma, Italy; Dip. Sc. Biomediche, Biotecnologiche e Traslazionali, University of Parma, Via A. Gramsci 14, 43126 Parma, Italy

**Keywords:** Cartilage, Chitosan, Rabbit

## Abstract

**Background:**

Osteochondral defects significantly affect patients’ quality of life and represent challenging tissue lesions, because of the poor regenerative capacity of cartilage. Tissue engineering has long sought to promote cartilage repair, by employing artificial scaffolds to enhance cell capacity to deposit new cartilage. An ideal biomaterial should closely mimic the natural environment of the tissue, to promote scaffold colonization, cell differentiation and the maintenance of a differentiated cellular phenotype. The present study evaluated chitosan scaffolds enriched with D-(+) raffinose in osteochondral defects in rabbits. Cartilage defects were created in distal femurs, both on the condyle and on the trochlea, and were left untreated or received a chitosan scaffold. The animals were sacrificed after 2 or 4 weeks, and samples were analysed microscopically.

**Results:**

The retrieved implants were surrounded by a fibrous capsule and contained a noticeable inflammatory infiltrate. No hyaline cartilage was formed in the defects. Although defect closure reached approximately 100% in the control group after 4 weeks, defects did not completely heal when filled with chitosan. In these samples, the lesion contained granulation tissue at 2 weeks, which was then replaced by fibrous connective tissue by week 4. Noteworthy, chitosan never appeared to be integrated in the surrounding cartilage.

**Conclusions:**

In conclusion, the present study highlights the limits of D-(+) raffinose-enriched chitosan for cartilage regeneration and offers useful information for further development of this material for tissue repair.

**Electronic supplementary material:**

The online version of this article (doi:10.1186/s12952-014-0021-5) contains supplementary material, which is available to authorized users.

## Background

Cartilage lesions represent a serious health concern because of the poor regenerative capacity of this tissue [1]. Cartilage damage leads to a natural repair process which mostly consists in the formation of fibro-cartilage, which lacks the strength and deformability characteristics of hyaline cartilage, with a consequent loss of joint functionality [2,3]. Tissue engineering approaches have long been tested to promote cartilage repair, by employing artificial substrates that can be used as a scaffold for cells, such as chondrocytes or autologous mesenchymal cells, capable to deposit new extracellular matrix [4,5]. The choice of a biomaterial with adequate characteristics is critical for clinical success [6], and an ideal biomaterial should closely mimic the natural environment of the tissue, to promote scaffold colonization, cell differentiation and the maintenance of the differentiated phenotype [7].

Chitosan is considered a promising material for the tissue engineering of articular cartilage [8,9] because of its similarity to glycosaminoglycans, important components of the extracellular matrix. Moreover, chitosan is degraded in vivo by lysozyme [10] and it has been hypothesized that catabolites from chitosan degradation could be involved in the synthesis of chondroitin sulfate and keratan. Consistently with this hypothesis, chitosan injections into the articular cavity of rats significantly increased chondrocyte density [11].

Chitosan properties can be affected by controlling the molecular weight of the monomer, its degree of deacetylation and its calcium content, which makes chitosan a very adaptable material to clinical requirements and needs [12]. Bettini and colleagues showed that raffinose at high concentrations makes the two-dimensional structure of chitosan smoother, highly hydrophilic, and with excellent elastic properties [13].

The purpose of the present study was to evaluate the effect of chitosan scaffolds enriched with D-(+) raffinose to promote the repair of cartilage defects in a rabbit model.

## Results

### Subcutaneous implant

**Figure 1:**
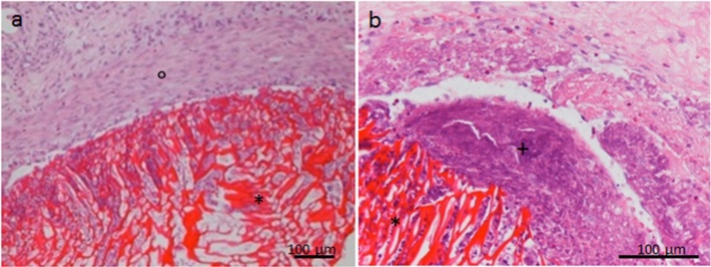
**Subcutaneous chitosan scaffold after 2 weeks.** Hematoxylin & Eosin staining, magnification 10X **(a)** and 20X **(b)**. Symbols: *scaffold,°fibrous capsule, + necrosis.

Histological examination of chitosan scaffolds inserted into a sub cute pouch in the inter-scapular region, showed that the material was properly positioned and surrounded by a fibrous capsule (Figure 1a). This capsule was homogeneous along the whole perimeter of the material, with limited areas of necrosis and inflammatory cells (Figure 1b).

The implant contained an inflammatory infiltrate comprising granulocytes, mostly neutrophils and eosinophils. Karyolysis, pyknosis and karyorrhexis were also observed. Noticeably, the number of fibroblasts infiltrating the outer pores of the scaffold was low.

### Osteochondral defects

Osteochondral defects were macroscopically evaluated by a 4-parameter assessment: joint functionality, defect contour, cartilage erosion and cartilage appearance. A final score was assigned by adding the scores for each tested parameter (Table 1). As for the first parameter, no animal showed signs of articular pain, indicating that the graft material did not affect joint function. The margins of the experimental defects were always clearly visible regardless of the presence of chitosan, even 4 weeks after surgery. As for cartilage erosion, cartilage was mostly intact around the defect, with one exception, where a little cartilage abrasion was observed around the trochlea in the presence of a chitosan implant. Almost all the cartilage defects however appeared eroded and damaged, both in the controls and in the chitosan-treated animals at all experimental time-points.

**Table 1:** **Data of the macroscopic score in the left side and data of O’Driscoll scoring system in the right side of the table**

**Sample**		**N**	**Mean**	**SD**	**Median**	**25 prcntil**	**75 prcntil**	**Sample**		**N**	**Mean**	**SD**	**Median**	**25 prcntil**	**75 prcntil**
Condilar Defect	Chitosan Scaffold 2W	9	2,78	0,67	3	2	3	Condilar Defect	Chitosan Scaffold 2W	9	4,78	1,92	4	3,5	6,5
	Surgical Control 2W	9	2,34	0,50	2	2	3		Surgical Control 2W	9	11,67	1,50	12	10,5	12
	Chitosan Scaffold 4W	9	2,56	0,53	3	2	3		Chitosan Scaffold 4W	9	4,78	1,30	4	4	6
	Surgical Control 4W	9	2,33	0,50	2	2	3		Surgical Control 4W	9	14,00	1,32	15	12,5	15
Trochlear Defect	Chitosan Scaffold 2W	9	3,40	1,13	3	2,5	4,5	Trochlear Defect	Chitosan Scaffold 2W	9	7,11	2,09	7	5	9,5
	Surgical Control 2W	9	3,00	0,87	3	2,5	3		Surgical Control 2W	9	9,78	3,15	10	7,5	12
	Chitosan Scaffold 4W	9	2,78	0,44	3	2,5	3		Chitosan Scaffold 4W	9	8,11	1,36	8	7	9,5
	Surgical Control 4W	9	4,11	0,93	4	3	5		Surgical Control 4W	9	13,78	1,09	14	13	15

**Figure 2:**
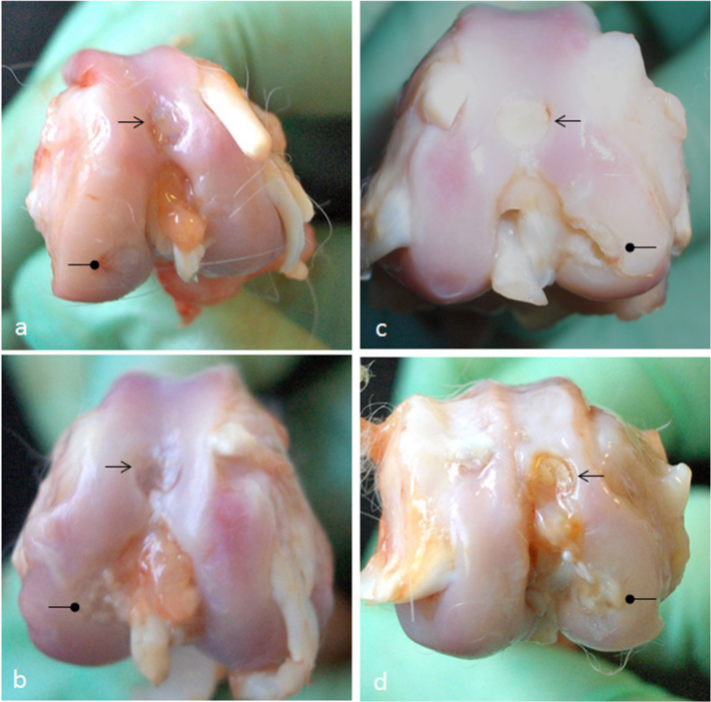
**Macroscopic view of the osteochondral defects in the surgical controls (a,b) and chitosan-treated defects (c,d) after 2 weeks (a,c) and 4 weeks (b,d).** Symbols: arrows indicate trochlear defects; rounded arrowheads indicate condylar defects.

**Figure 3:**
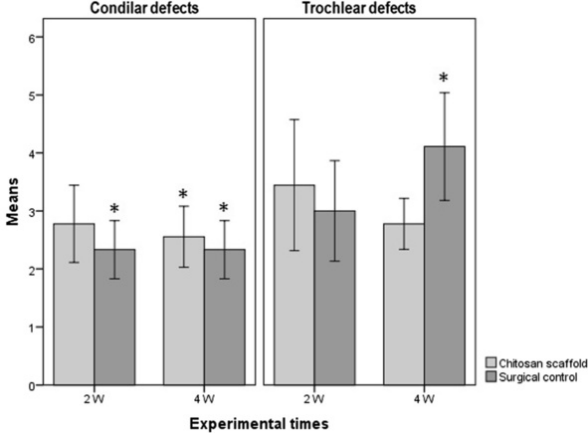
**Macroscopic evaluation of the osteochondral defects expressed as mean ± standard deviation.** 2W (2 weeks), 4W (4 weeks). * indicates statistical significance.

After 2 and 4 weeks, almost all the condylar defects appeared visually colourless and irregular, both in the control and in the chitosan-treated group (Figure 2). Noteworthy, trochlear defects appeared matt in all animals, and no observer found the presence of translucent, cartilage-like tissue. No significant difference was found in the overall score between the groups (Figure 3), with the only exception of the control trochlear implant at 4 weeks. The score for this site was significantly higher than that of the control trochlear at 2 weeks (p ≤ 0.05), control condyle at 4 weeks (p ≤ 0.01), control condyle at 2 weeks (p ≤ 0.001), treated trochlea at 4 weeks (p ≤ 0.01), treated condyle at 4 weeks (p ≤ 0.01), treated condyle at 2W (p ≤ 0.01).

### Scaffold porosity evaluation

Image analysis was used to assess the effects of scaffold and implant site on wound healing. Although all the scaffolds were placed inside the surgical defects, scaffold deformation, resulting in oval or bilobate shapes, was often observed, and this may have affected the contact between the scaffold and surrounding tissue.

**Figure 4:**
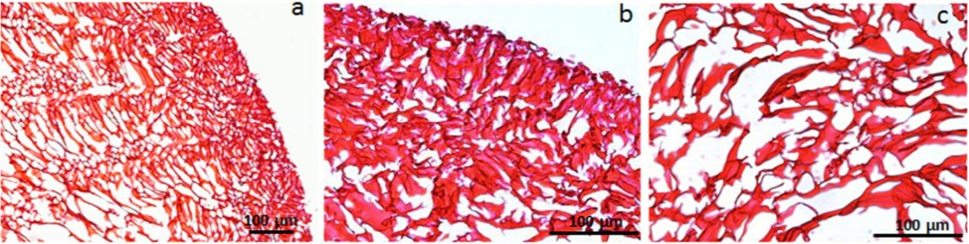
**Microphotographs of pre-grafting chitosan scaffold with Hematoxylin & Eosin staining.** Magnification 10X **(a)** and 20X **(b, c)**.

**Figure 5:**
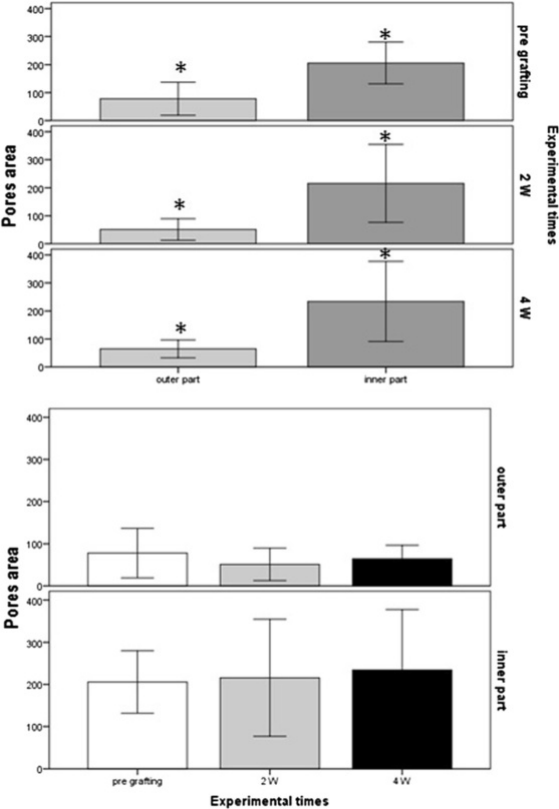
**Scaffold porosity evaluation.** Pore area is expressed as mean ± standard deviation (μm^2^). 2W (2 weeks), 4W (4 weeks), *indicate statistical significance.

Scaffold porosity was evaluated by measuring pore area (μm^2^) in the outer circumferential part of the chitosan implant and in the inner portion (Figure 4). Outer pores were significantly smaller than inner pores (Figure 5), both in the pre-grafting scaffold (outer pore area M = 77.00 ± 58.97; area of the internal pores M = 205.80 ± 74.25) and after grafting at both experimental time points. The outer pore area was 51.15 ± 38.63, and the inner pore area was 215.67 ± 138.88 at 2 weeks, whereas the outer pore area was 64.45 ± 31.89 and the inner pore area was 234.16 ± 143.10 at 4 weeks (p <0.01).

### Histologic analysis of defects

Microscopic analysis was performed using the O’Driscoll scoring test, to understand the quality of the newly deposited tissue (Table 1).

**Figure 6:**
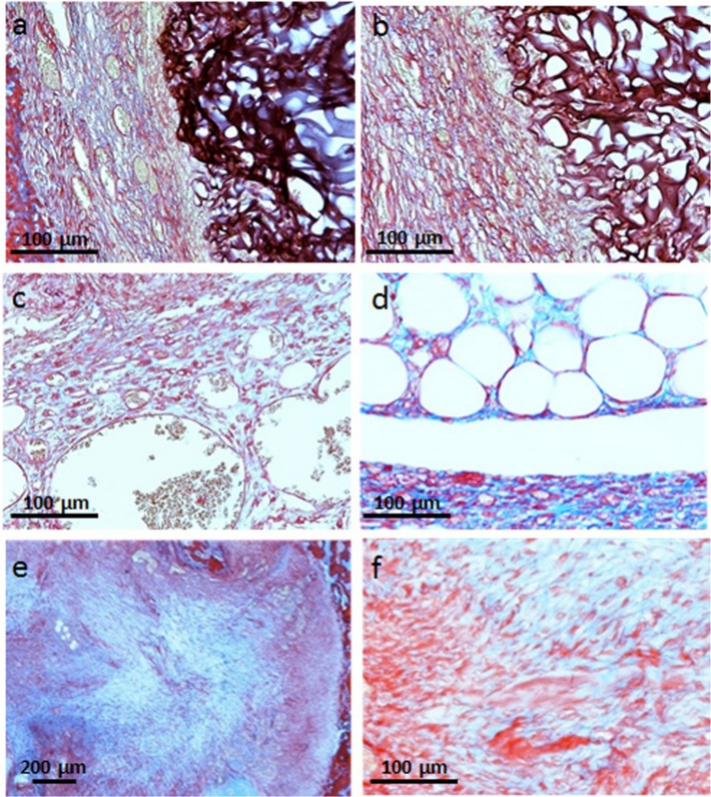
**Histology microphotographs of the defects stained with Masson’s trichrome.** Chitosan scaffold treated defects **(a,b,c,d)** after 2 weeks **(a,c,d)** and 4 weeks **(b)**. Surgical controls after 4 weeks **(e,f)**.

Sample analysis revealed that no hyaline cartilage formed in the osteochondral defects, but only a granulation tissue and fibrous cartilage were observed. This finding was confirmed by Masson staining, which highlighted the absence of glycosaminoglycans typical of hyaline cartilage. Defect closure reached approximately 100% in the control group after 4 weeks (Figure 6e, f). However, defects did not completely heal when filled with chitosan. The newly formed tissue was localized only in the most superficial part of the scaffolds and in the gap that was created by scaffold deformation (Figure 6d). Two weeks after surgery, the newly deposited tissue appeared to be exclusively composed of granulation tissue (Figure 6a), with numerous fibroblasts near the blood vessels (Figure 6c). By 4 weeks the granulation tissue started to be replaced by a fibrous connective tissue, both in the condyle and in the trochlea. The interface between the defect and the surrounding tissue appeared to possess a more solid and compact structure in the control group as compared to chitosan-treated defects. Chitosan did not appear firmly anchored to the defect regardless of time point and implant site, and the newly formed connective tissue presented with only a scant and superficial bond with the surrounding cartilage (Figure 6b).

**Figure 7:**
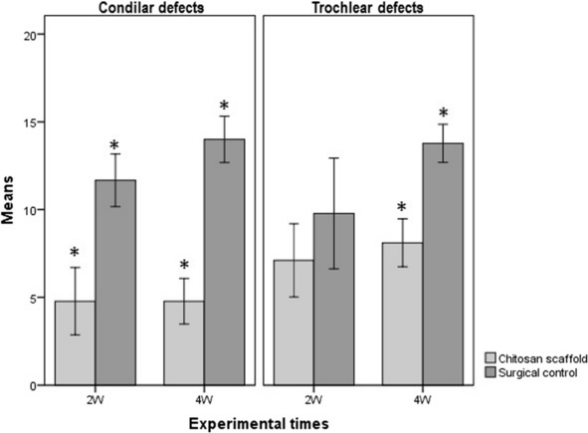
**Results of the microscopic analysis performed using the O’Driscoll scoring expressed as mean ± standard deviation.** 2W (2 weeks), 4W (4 weeks), *indicates statistical significance.

The results from the O'Driscoll test are reported in Figure 7. As for condyle defects, treated animals had a lower score compared to controls at 2 weeks (p < 0.01) and at 4 weeks (p < 0.001).

A lower score was also recorded in chitosan-treated trochlear defects than in controls at 2 weeks of treatment, and this trend was confirmed at 4 weeks as well (p < 0.001). Treated defect score did not significantly change over time for both condyle and trochlea.

### Polarised light microscopic analysis

**Figure 8:**
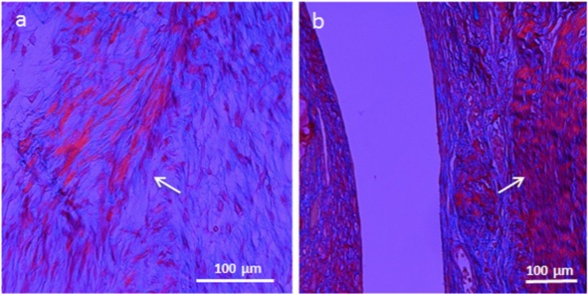
**Microphotographs of histological sections at polarized light.** Arrows indicate birefringent collagen bundles in surgical control **(a)** and chitosan treated defect **(b)**.

A poorly organised fibrocartilage tissue was observed 2 weeks after surgery in control condylar defects with the presence of only a small amount of birefringent collagen fibers (Figure 8). Fibroblasts were also visible around the defect. No birefringent

collagen bundles were detectable in the treated samples and cell arrangement was not suggestive of any real cellular organization. As for trochlear defects, collagen bundles were observed only in the control group, but were absent in the chitosan-treated defects. After 4 weeks, fibrocartilage tissue completely filled the control defects, with well evident birefringent collagen fibers within the newly formed tissue.

When chitosan was implanted in the defects, although fibrocartilage was observed (Figure 8), it did not fill the whole defect and still presented with a poorly organised structure.

## Discussion

The present work focused on the possible use of D-(+) raffinose-enriched chitosan for cartilage regeneration and offers some insight on the use of this biopolymer as scaffold in cartilage defects [6,8,12,14,15]. Histological examination of D-(+) raffinose-enriched chitosan implanted into the sub cute indicated that the graft material stimulated fibroblast proliferation, which created a fibrous capsule around the material. An abundant inflammatory infiltrate, mainly consisting of granulocytes, was observed inside the chitosan mesh. The presence of these cells in chitosan scaffolds has already been described and some authors demonstrated a chemotactic effect by this material on inflammatory cells [16-19]. Neutrophil migration appears to be associated to specific interactions between chitosan oligosaccharide and surface receptors on the membrane of neutrophils, and to depend on chitosan chemical modifications [20]. Neutrophil infiltration is an undesired event, as increased inflammation has long been associated to poor implant integration [21]. Moreover, the presence of limited necrotic areas in the outermost layer of the implanted material raises questions on the biocompatibility of chitosan modified with D-(+) raffinose. However, recent studies have shown that chitosan interactions with the host’s immune system is complex and involves the modulation of anti-inflammatory cell and cytokine systems [22].

Strikingly, the macroscopic observation indicated that the repair process was not affected by the presence of chitosan and microscopic analysis confirmed that defect healing was not complete in the presence of the scaffold. The natural process of cartilage repair, as visible in the surgical controls, involved the formation of fibrocartilage, which does not possess comparable mechanical properties to hyaline cartilage, and thus causes a decrease in joint function. The presence of scaffold remnants inside the defects even after 4 weeks of healing was not unexpected, as the chitosan used in this experiment had a very high degree of deacetylation (92.8%), which significantly affected its rate of resorption. This finding is consistent with data reported in the literature, and actually suggests that the greater the degree of deacetylation of chitosan and the lower its resorption rate [12].

Microscopic evaluation showed that the material did not maintain its original cylindrical shape throughout the experiment, but significant deformations were observed. This created gaps, which reduced the scaffold-tissue contact, and presumably the scaffold effectiveness to promote tissue regeneration. It can be assumed that this phenomenon was caused by the high deformability of this material together with the surgical protocol used in the study, and less deformable scaffolds will have to be tested to verify this hypothesis. The formation of gaps, together with the different degree of porosity found throughout the scaffold and the absence of vascularization are the main obstacles to scaffold colonization that emerged in this study. The different degree of porosity observed between the outer material and the inner part, may have affected the colonization of the deeper areas of the scaffolds, because the reduced average area of the outer pores may have prevented cell migration into the scaffold. A work by Lien and colleagues suggests that the optimal size of the pores is closer to that found in the inner part the scaffolds [23]. Consistently with this phenomenon, microscopic analysis revealed the absence of vascular structures in chitosan at both experimental time points, which arguably represents a limiting factor for the colonization of the inner portion of the scaffolds, as this affected scaffold perfusion and the supply of nutrients and oxygen [24]. Vessels however were abundant inside the granulation tissue around the scaffold, as expected [8,12]. In fact it seems that chitosan induced a transient vascularization when implanted into cartilage [14,25].

The observed difference in scaffold porosity was not caused by scaffold compression upon insertion in the defect, as it was already present in pristine samples, and it is most likely to be attributed to the synthesis procedure. As a consequence, the preparation of the scaffolds will have to be modified to allow for larger pores on the outer surface.

Moreover, differences in the structure of the newly formed tissue were observed when samples in the presence or in the absence of mechanical loading were compared. A layer of connective tissue bridging the gap, thus providing an early, albeit non-functional structural support, was observed in samples inserted in the condyles. Polarized-light histology revealed that this was an areolar connective tissue with a random distribution of collagen bundles. When chitosan was inserted in the trochlea however, a dense irregular connective tissue was deposited. The higher density of the tissue was likely due to a greater degree of internal structure of the tissue, possibly because of the absence of a physiological load. Consistently with this hypothesis, the polarized light showed that the collagen bundles were mostly located circumferentially around the defect.

## Conclusions

In conclusion, the present preliminary study offers some useful information for further experiments aimed at optimizing the performance of chitosan enriched with D-(+) raffinose in cartilage repair, as it highlighted several shortcomings of these scaffolds, which need to be corrected to improve their performance. Changes in material preparation can be envisaged to reduce its deformation thus increasing its contact with the surrounding tissue and to promote cellular colonization by increasing pore size in its outer portion. These scaffold could also be loaded with autologous adult or mesenchymal stem cells, or with growth or adhesion factors prior to implantation [15,26] to enhance cell migration.

## Methods

### Animals

Male New Zealand White rabbits (Harlan Laboratories, Correzzana, Italy) were used for the study. The national and European legislation regarding the protection of animals used for experimental purposes (European Union 2010/63 – Health Ministry Authorization 16/04/2010) was strictly followed in all experimental phases. Since the amount of the effect due to the chitosan scaffold was unknown, the present study can be considered as “pilot” experience. The number of animals involved were calculated on the basis of the ethical aspects imposed by international and national regulations (European union 2010/63 – National DLgs 26/2014), which recommend to minimize the number of individuals for experimental trials and on the basis of the authors' experience with this animal model.

The experimental plan (University of Parma Ethical Committee approval 19/01/2007) involved the use of seven male rabbits from same litters, weighing 4.2 ± 0.2 kg. Animals were housed in separate cages, at a temperature of 20 ± 2°C, with a 12 hours light and 12 hours dark photoperiod, and were fed ad libitum with commercial pelleted feed. Drinking water was administered ad libitum.

### Surgeries

The implant site was the femur distal epiphysis, which is covered by articular cartilage. The rabbits underwent surgeries a week after their arrival from the vendor. Rabbits were anesthesized according to an approved protocol (Domitor, Pfizer, 0.1 ml/kg; Ketavet 100, Gellini, 0.3 ml/kg; Isoflurane-Vet, Mérial). The surgical protocol included patellar arthrotomy with medial dislocation of the patella to expose the articular surface of the femur. Two osteochondral defects were created in each femur, one on the medial condyle and the other on the trochlea, respectively, to mimic loading and non loading conditions. The osteochondral defects were created with a low speed surgical drill, along the major axis of the femur, under continuous irrigation with saline solution. The defects had a diameter of 2.7 mm and a depth of about 5 mm. The defects in the right segment received chitosan scaffolds of 2.6 mm diameter and 5 mm in length, while the defects on the left segment were left untreated and served as control. The flap was repositioned after implantation, the joint capsule and the soft tissues were sutured (Assufil 3/0, Assut Sutures, Pully-Lausanne Switzerland). A subcutaneous inter scapular pocket was created for one animal, and a chitosan scaffold was inserted. The skin was then sutured as described above. The animals were randomly assigned to two groups consisting of three animals each, for sacrifice at 2 and 4 weeks (respectively 2W and 4W). All animals were subjected to a complete clinical examination and orthopedic examination on a weekly basis, to assess the presence of swelling, movement impairment, pain during passive movement and limb functional recovery. The animal with the dorsal sub cute implant underwent weekly clinic visits to assess the presence of inflammation at the insertion site.

### Sample collection and processing

The animals were sacrificed with i.p. injections (Tanax, Roussel Hoechst Agrovet, 0.3 ml/kg) after general anesthesia (Prequillan and Ketavet). The limbs were excised and soft tissues were removed, to expose the knee joint and allow for sample harvesting. The samples were fixed in 10% formaldehyde for 24 hours, washed in running water and decalcified with an EDTA-based solution (Microdec based EDTA, Diapath, Martinengo, Italy) for 72 hours. They were then dehydrated in alcohols at increasing concentrations (Carlo Erba reagents), cleared in xylene (Carlo Erba Reagents, Cornaredo, Italy) for two hours and impregnated in liquid paraffin (Bio-plast plus, Bio-Optica, Milan, Italy) for about two hours. The procedure was performed with a tissue processor (ATP 700 Tissue Processor, Histo-line, Milan, Italy). At the end of this procedure, the samples were embedded in paraffin (Bio-plast plus, Bio-Optica) with a processor (TBS88, Medite, Burgdorf, Germany) and perpendicular sections were obtained (RM 2155, Leica) . Sections were stained with hematoxylin-eosin to assess cellular distribution and morphology. Samples from the osteochondral defects were also stained with Masson's trichrome staining (Kit, Bio-Opitca) (64), whereas sub cute samples were also stained with Giemsa staining. Each sample was evaluated by three different authors and scored as described below.

### Macroscopic defect evaluation

The knee joint was macroscopically evaluated after exposure, using a validated scoring system [27]. The score ranged from a minimum value equal to 0 and a maximum value of 2. The test was modified because it was impossible to assess the degree of intra-articular fibrosis. The considered parameters were the degree of movement as assessed by joint mobility, defect outline, whether the margins were still distinguishable, cartilage erosion, cartilage appearance, whether it appeared translucent, as typical in hyaline cartilage, matte or colourless and irregular, as in fibrocartilage.

### Histological analysis

The histological analysis (with O’Driscoll scoring systems) and the acquisition of images in polarized light, have been carried out by means of a motorized microscope (Eclipse 90i, Nikon, Tokyo, Japan), equipped with a digital camera (Nikon model DS-5M) and equipped with image analyzer (NIS-Advanced Reserarch 2.1, Nikon).

### Microscopic evaluation

The microscopic analysis was carried out using O'Driscoll scoring system [28], modified using Masson's trichrome staining. The parameters taken into consideration were the predominant nature of the neo-formed tissue, whether hyaline cartilage, cartilage or undifferentiated tissue, fibrous tissue or bone. Furthermore, cellularity and structural characteristics such as surface regularity, structural integrity, the percentage of defect closure and the union with the adjacent tissue, were considered.

### Scaffold analysis

Porosity analysis was carried out with an image analyzer (NIS-Advanced Reasearch 3.1, Nikon). For analysis purposes, the scaffold was divided into an outer and an inner circumferential portion. Pore area, expressed in μm^2^, was measured both pregrafting and after grafting at both experimental time points (2W and 4W).

### Statistical analysis

Data were analyzed using statistical software SPSS 18 (SPSS inc., IBM) and reported in Table 1. For the macroscopic and microscopic scoring system the Kruskal-Wallis analysis followed by a post-hoc Mann–Whitney pairwise comparison test was used. 95% confidence intervals of estimated differences between means were calculated for each comparison. For the scaffold porosity the analysis of variance ( ANOVA) followed by post test (Tukey, Scheffe) was used. Results were considered statistically significant when p < 0.05.

## Abbreviations

SD: Standard deviation
2W: 2 weeks
4W: 4 weeks

## References

[1] Chung R, Xian CJ (2014). Recent research on the growth plate: mechanisms for growth plate injury repair and potential cell-based therapies for regeneration. J Mol Endocrinol.

[2] Pearle AD, Warren RF, Rodeo SA (2005). Basic science of articular cartilage and osteoarthritis. Clin Sports Med.

[3] Moran CJ, Pascual-Garrido C, Chubinskaya S, Potter HG, Warren RF, Cole BJ (2014). Restoration of articular cartilage. J Bone Joint Surg Am.

[4] Saha S, Kirkham J, Wood D, Curran S, Yang XB (2013). Informing future cartilage repair strategies: a comparative study of three different human cell types for cartilage tissue engineering. Cell Tissue Res.

[5] Shimomura K, Moriguchi Y, Murawski CD, Yoshikawa H, Nakamura N (2014). Osteochondral Tissue Engineering with Biphasic Scaffold: Current Strategies and Techniques. Tissue Eng Part B Rev.

[6] Nettles DL, Elder SH, Gilbert JA (2002). Potential use of chitosan as a cell scaffold material for cartilage tissue engineering. Tissue Eng.

[7] Lim EH, Sardinha JP, Myers S (2014). Nanotechnology biomimetic cartilage regenerative scaffolds. Arch Plast Surg.

[8] Suh JK, Matthew HW (2000). Application of chitosan-based polysaccharide biomaterials in cartilage tissue engineering: a review. Biomaterials.

[9] Khan F, Ahmad SR (2013). Polysaccharides and their derivatives for versatile tissue engineering application. Macromol Biosci.

[10] Xia W, Liu P, Liu J (2008). Advance in chitosan hydrolysis by non-specific cellulases. Bioresour Technol.

[11] Lu JX, Prudhommeaux F, Meunier A, Sedel L, Guillemin G (1999). Effects of chitosan on rat knee cartilages. Biomaterials.

[12] Abarrategi A, Lopiz-Morales Y, Ramos V, Civantos A, Lopez-Duran L, Marco F (2010). Chitosan scaffolds for osteochondral tissue regeneration. J Biomed Mater Res A.

[13] Bettini R, Romani AA, Morganti MM, Borghetti AF (2008). Physicochemical and cell adhesion properties of chitosan films prepared from sugar and phosphate-containing solutions. Eur J Pharm Biopharm.

[14] Hoemann CD, Hurtig M, Rossomacha E, Sun J, Chevrier A, Shive MS (2005). Chitosan-glycerol phosphate/blood implants improve hyaline cartilage repair in ovine microfracture defects. J Bone Joint Surg Am.

[15] Chen YL, Chen HC, Chan HY, Chuang CK, Chang YH, Hu YC (2008). Co-conjugating chondroitin-6-sulfate/dermatan sulfate to chitosan scaffold alters chondrocyte gene expression and signaling profiles. Biotechnol Bioeng.

[16] Usami Y, Okamoto Y, Takayama T, Shigemasa Y, Minami S (1998). Chitin and chitosan stimulate canine polymorphonuclear cells to release leukotriene B4 and prostaglandin E2. J Biomed Mater Res.

[17] VandeVord PJ, Matthew HW, DeSilva SP, Mayton L, Wu B, Wooley PH (2002). Evaluation of the biocompatibility of a chitosan scaffold in mice. J Biomed Mater Res.

[18] Vasconcelos DP, Fonseca AC, Costa M, Amaral IF, Barbosa MA, Aguas AP (2013). Macrophage polarization following chitosan implantation. Biomaterials.

[19] Almeida CR, Serra T, Oliveira MI, Planell JA, Barbosa MA, Navarro M (2014). Impact of 3-D printed PLA- and chitosan-based scaffolds on human monocyte/macrophage responses: unraveling the effect of 3-D structures on inflammation. Acta Biomater.

[20] Park CJ, Gabrielson NP, Pack DW, Jamison RD, Wagoner Johnson AJ (2009). The effect of chitosan on the migration of neutrophil-like HL60 cells, mediated by IL-8. Biomaterials.

[21] Griffiths MM, Langone JJ, Lightfoote MM (1996). Biomaterials and granulomas. Methods.

[22] Oliveira MI, Santos SG, Oliveira MJ, Torres AL, Barbosa MA (2012). Chitosan drives anti-inflammatory macrophage polarisation and pro-inflammatory dendritic cell stimulation. Eur Cell Mater.

[23] Lien SM, Ko LY, Huang TJ (2009). Effect of pore size on ECM secretion and cell growth in gelatin scaffold for articular cartilage tissue engineering. Acta Biomater.

[24] Woo Jung J, Yi HG, Kang TY, Yong WJ, Jin S, Yun WS (2013). Evaluation of the effective diffusivity of a freeform fabricated scaffold using computational simulation. J Biomech Eng.

[25] Chevrier A, Hoemann CD, Sun J, Buschmann MD (2007). Chitosan-glycerol phosphate/blood implants increase cell recruitment, transient vascularization and subchondral bone remodeling in drilled cartilage defects. Osteoarthritis Cartilage.

[26] Lee JE, Kim KE, Kwon IC, Ahn HJ, Lee SH, Cho H (2004). Effects of the controlled-released TGF-beta 1 from chitosan microspheres on chondrocytes cultured in a collagen/chitosan/glycosaminoglycan scaffold. Biomaterials.

[27] Xie J, Han Z, Naito M, Maeyama A, Kim SH, Kim YH (2010). Articular cartilage tissue engineering based on a mechano-active scaffold made of poly(L-lactide-co-epsilon-caprolactone): in vivo performance in adult rabbits. J Biomed Mater Res B Appl Biomater.

[28] O'Driscoll SW, Keeley FW, Salter RB (1988). Durability of regenerated articular cartilage produced by free autogenous periosteal grafts in major full-thickness defects in joint surfaces under the influence of continuous passive motion: a follow-up report at one year. J Bone Joint Surg Am.

